# Mixed neurotransmission in the hippocampal mossy fibers

**DOI:** 10.3389/fncel.2013.00210

**Published:** 2013-11-22

**Authors:** Agnieszka Münster-Wandowski, Gisela Gómez-Lira, Rafael Gutiérrez

**Affiliations:** ^1^Institute of Integrative Neuroanatomy, Charité-Universitätsmedizin BerlinCampus Mitte, Berlin, Germany; ^2^Department of Pharmacobiology, Centro de Investigación y Estudios Avanzados del Instituto Politécnico NacionalMexico City, Mexico

**Keywords:** mossy fibers, dentate gyrus, GABA, co-release, gap junctions, CA3

## Abstract

The hippocampal mossy fibers (MFs), the axons of the granule cells (GCs) of the dentate gyrus, innervate mossy cells and interneurons in the hilus on their way to CA3 where they innervate interneurons and pyramidal cells. Synapses on each target cell have distinct anatomical and functional characteristics. In recent years, the paradigmatic view of the MF synapses being only glutamatergic and, thus, excitatory has been questioned. Several laboratories have provided data supporting the hypothesis that the MFs can transiently release GABA during development and, in the adult, after periods of enhanced excitability. This transient glutamate-GABA co-transmission coincides with the transient up-regulation of the machinery for the synthesis and release of GABA in the glutamatergic GCs. Although some investigators have deemed this evidence controversial, new data has appeared with direct evidence of co-release of glutamate and GABA from single, identified MF boutons. However, this must still be confirmed by other groups and with other methodologies. A second, intriguing observation is that MF activation produced fast spikelets followed by excitatory postsynaptic potentials in a number of pyramidal cells, which, unlike the spikelets, underwent frequency potentiation and were strongly depressed by activation of metabotropic glutamate receptors. The spikelets persisted during blockade of chemical transmission and were suppressed by the gap junction blocker carbenoxolone. These data are consistent with the hypothesis of mixed electrical-chemical synapses between MFs and some pyramidal cells. Dye coupling between these types of principal cells and ultrastructural studies showing the co-existence of AMPA receptors and connexin 36 in this synapse corroborate their presence. A deeper consideration of mixed neurotransmission taking place in this synapse may expand our search and understanding of communication channels between different regions of the mammalian CNS.

## INTRODUCTION

By the end of the 1800s and early 1900s, research on the nervous system was directed toward defining its conformation, particularly, to answer whether it was constituted by individual entities, neurons (neuronal doctrine championed by Santiago Ramón y Cajal) or by a continuous network (reticular hypothesis, championed by Camilo Golgi).

The debate was even present in the Nobel Lectures, held by those who shared the prize in 1906: Ramón y Cajal (1906) and Golgi (1906), both “in recognition of their work on the structure of the nervous system.” The neuronal doctrine prevailed as evidence accumulated, but this issue was not only solved by anatomists. Indeed, physiologists played an important role in defining the concept of neuron while they were discussing about another related issue: whether transmission among nerves and effector organs/muscles was chemical or electrical. The growing evidence in favor of chemical transmission, in turn, supported the growing anatomical evidence of neurons being independent entities.

The groundbreaking work of Loewi and Dale, who demonstrated that stimulation of the nerves of the autonomic nervous system produced the release of “substances” that had inhibitory or excitatory effects on their target organs, established chemical transmission as a “general principle.” However, during the 1930s to the 1950s several electrophysiologists, including most notoriously Eccles, argued that in nicotinic synapses the excitation of the nerves generated a current that flowed into the postsynaptic cell and that it could be followed by a residual action mediated by a chemical transmitter such as acetylcholine (Ach). Doubts were dissipated and it became clear that peripheral synapses were of chemical nature, mainly through the work of Bernard Katz and coworkers. Eccles, despite his finally endorsing the chemical synapse in ganglia said: “⋯it would seem expedient to restrict it (the electrical synapse) in the first instance to monosynaptic transmission through the spinal cord, where chemical transmission by ACh seems highly improbable, and where the experimental investigation has been more rigorous than elsewhere in the nervous system” (cited in Cowan et al., 2001). This view, or rather defense of the electrical synapse, prevailed until he and his group discovered synaptic inhibition at the beginning of the 1950s, of course of chemical nature, but the transmitters involved were still to be discovered. So, at that stage, a key question was still open. Are there more chemicals in the central nervous system (CNS) than adrenalin and ACh, shown to act in the autonomic nervous system and, the latter, also in the neuromuscular junction? One thing seemed to be clear: that neurons released what they produced and contained. And, in this context, Dale (1935) concluded that a neuron functions as a metabolic unit, therefore, a process occurring in the cell influences all of the compartments of that given neuron. In other words, the neuron would release the same neurotransmitter from all its terminals. Unfortunately, this was transformed in the literature to a “principle” stating that “a single cell releases only one neurotransmitter” or that “its action is the same on all its postsynaptic targets.”

A whole range of transmitter substances were discovered during the last half of the twentieth century, ranging from amino acids (glutamate, GABA, glycine, etc.) to dopamine (DA), norepinephrine and serotonin and, finally, a long list of neuropeptides. On the other hand, in the 1970s it became evident that the “one neuron, one neurotransmitter” postulate is the exception rather than the rule because many of these substances co-exist and can be simultaneously released from single cells and even segregated so as to be released from different terminals of the same cell. Therefore, it was established that a cell could co-release a fast-acting “classical” neurotransmitter and a modulatory factor, that could be a peptide, nucleotide (e.g., ATP), Zn^2+^, neurotrophic factor, nitric oxide, or endogenous cannabinoids – among other modulators. By contrast, the co-existence and co-release of two or more “classical” neurotransmitters, each conveying a “principal message,” has been less studied. However, during recent years, several research groups have shown that co-release is not that uncommon, and it is now known to occur in a variety of neural systems. In addition, although many populations of adult neurons may not release two classical neurotransmitters under basal conditions, many appear to do so transiently following an established program during early development, or in response to a variety of physiological and pathophysiological stimuli (Gutiérrez, 2009; Dulcis and Spitzer, 2011; Borodinsky et al., 2012).

But what about “co-existence” of electrical and chemical transmission? After years of efforts to prove the chemical nature of synaptic transmission in the CNS, Fatt (1954) pointed out that it was premature to conclude that chemical transmission was in fact universal. Soon enough, Furshpan and Potter (1957) showed that the passage of electrical current accounted for the transmission of information between the presynaptic giant axon and the post-synaptic motor axon in the crayfish nerve cord. Moreover, it was found that electrical communication can also cause inhibition (Furukawa and Furshpan, 1963). The work of Bennett and collaborators established that in most cases the flow of current in electrical synapses can be in either direction and that they were most commonly found in rapidly activated circuits, where reciprocity and speed are important for the synchrony of ensembles of neurons (Bennett, 1972). Finally, Martin and Pilar (1963) discovered that both electrical and chemical communication do coexist in the chick ciliary ganglion. It is now recognized that electrical communication through gap junctions in dendro-dentritic and axo-axonic synapses is more common than originally thought in the mammalian brain (Connors and Long, 2004; Söhl et al., 2005). By contrast, although already detected (for a review see Connors and Long, 2004), mixed electrical-chemical communication involving axo-dendritic contacts is still under scrutiny.

In line with this introduction concerning mixed synaptic transmission, in this review we examine the surprising physiology of the mossy fibers (MFs). Indeed, these axons have proven to be neuronal elements still full of surprises. The traditional working paradigm of those working on MF transmission is that granule cells (GCs) form glutamatergic synapses with pyramidal cells and local inhibitory interneurons of the CA3 area. Thus, activation of the MF provokes monosynaptic excitatory responses in both cell types, and the activation of interneurons, in turn, produces inhibitory responses in pyramidal cells to maintain feedforward inhibition (Lawrence and McBain, 2003). Therefore, activation of GC during the blockade of glutamate-mediated transmission prevents both excitatory and inhibitory responses from appearing in CA3. However, two sets of data that add a twist to this paradigm will be the focus of this review: the possibility of GCs co-releasing glutamate and gamma-aminobutyric acid (GABA) on to their target cells in CA3 area and the possibility of some MFs establishing mixed electrical-chemical synapses among them and some establishing axo-dendritic mixed, electrical-chemical synapses with CA3 pyramidal cells.

## DEFINING MARKERS OF GLUTAMATERGIC AND GABAERGIC NEURONS

Glutamatergic and GABAergic neuronal phenotypes have long been considered independent, each being characterized by dedicated molecular components or markers that have a segregated cellular and subcellular localization. However, recent research has challenged these long-standing assumptions, whereby the co-localization of glutamatergic and GABAergic molecular components has been found in both, glutamatergic and GABAergic cells in various brain regions, most notoriously in dentate GCs and the MF system. What does a glutamatergic or a GABAergic cell contain or express to be defined as such?

### GLUTAMATE

Glutamate is the major, widely distributed, excitatory neurotransmitter in the mammalian CNS. In addition to its involvement in fast synaptic transmission, it has a role in long-term potentiation, as a proposed molecular substrate for learning and memory (Attwell, 2000). Glutamine and alpha-ketoglutarate, the major precursors of glutamate, are actively taken up into the pre-synaptic terminal via an active, Na^+^-dependent uptake mechanism (Anderson and Swanson, 2000; Daikhin and Yudkoff, 2000). Glutamine is then transported to mitochondria, where it is converted via phosphate-activated glutaminase to glutamate and ammonia. Alternatively, glutamate may be formed from alpha-ketoglutarate and alanine catalyzed by alanine aminotransferase. The cytoplasmic glutamate is packed into synaptic vesicles by vesicular glutamate transporters (VGLUTs) for future release into the synaptic cleft by a process of vesicular exocytosis (Südhof et al., 1993; Bennett and Scheller, 1994; Söllner and Rothman, 1994). Glutamate is rapidly removed from the cleft by being: (1) actively transported back to the neuron and the glial cells or (2) inactivated by enzymatic degradation (Anderson and Swanson, 2000; Attwell, 2000; Daikhin and Yudkoff, 2000). Glutamate uptake is mediated by plasma membrane glutamate transporters EAAC1/EAAT3 and GLT1/EAAT2, respectively.

In glial cells, glutamate is metabolized to either glutamine via glutamine synthase or α-ketoglutarate by glutamate oxaloacetate transaminase or glutamate dehydrogenase (Meldrum et al., 1999; Anderson and Swanson, 2000). These two precursors, glutamine and α-ketoglutarate are then actively transported out of the glial cells and back into the pre-synaptic axon terminals for subsequent re-synthesis of glutamate (Meldrum et al., 1999).

In GABAergic interneurons glutamate can be synthesized via a different enzymatic route, involving glutamate dehygrogenase (Schmitt and Kugler, 1999), aspartate aminotransferase (Rothe et al., 1995), and glutaminase (Laake et al., 1999; Holten and Gundersen, 2008). This is in line with the fact that there is a significant level of glutamate in GABA nerve terminals, as shown by electron microscopic immunogold technique (Laake et al., 1999; Holten and Gundersen, 2008).

The concentration of glutamate within the vesicle is thought to be ~100 mmol/L. Release of a single vesicle is believed to produce high concentrations in the cleft to saturate postsynaptic receptors and induce a “quantal-sized” excitatory post-synaptic potential (EPSP) (Meldrum, 2000). The extracellular concentration of glutamate *in vivo* is normally very low (~1 μM; Anderson and Swanson, 2000; Attwell, 2000), enough to prevent excitotoxicity (Choi, 1992; Rothstein et al., 1996).

### GABA

In the mammalian CNS, inhibition is primarily mediated by the amino acids GABA and glycine. GABA is the main inhibitory neurotransmitter in the adult cortex and its synthesis is unique among neurotransmitters, having two separate isoforms of the rate-controlling enzyme, glutamic acid decarboxylase GAD65 and GAD67, that convert excitatory amino acids into GABA. These isoforms differ in their cellular localization and functional properties. GABA synthesized by GAD65 is used for neurotransmission whereas GABA synthesized by GAD67 is used for processes such as synaptogenesis and protection against neuronal injury. GABA is loaded into synaptic vesicles with the help of the vesicular GABA transporter (VGAT) by using the electrochemical gradient of H^+^ (Ahnert-Hilger and Jahn, 2011; Riazanski et al., 2011). Additionally, chloride gradients between the vesicle lumen and the presynaptic cytosol may contribute to the vesicular loading of GABA (Ahnert-Hilger and Jahn, 2011; Riazanski et al., 2011). GAD65 and VGAT are functionally linked at the synaptic vesicle membrane and GABA synthesized by GAD65 is preferentially loaded into the synaptic vesicle over GABA synthesized in cytoplasm by GAD67. In a GABAergic synapse it is likely that approximately 80% of the released GABA is transported back into the GABAergic nerve ending, whereas the remaining GABA will be taken up into the astrocytes in the proximity of the synapse (Hertz and Schousboe, 1987).

There have been no functional estimates of intracellular GABA levels until now. Previous studies proposed GABA concentrations ranging from 7 mM in somata (Obata et al., 1970; Otsuka et al., 1971) to 50–150 mM in nerve terminals (Fonnum and Walberg, 1973). These estimates raise the intriguing possibility that VGAT saturation is a prerequisite for efficient refilling of inhibitory vesicles. However, Apostolides and Trussell (2013) found that the endogenous transmitter does not saturate vesicular transporters: dialyzing presynaptic cells with high concentrations of glycine or GABA reduced the efficacy of a fast-off glycine receptor antagonist and potentiated GABAergic transmission.

### GLUTAMATE AND GABA TRANSPORTERS

Glutamate and GABA transporters play a major role in the removal of transmitters from the synaptic cleft and the extracellular space, recycling, and repackaging them in synaptic vesicles. On the basis of their structure and site of action, transporters can be divided into two superfamilies: (1) vesicular transporters and (2) plasma membrane transporters.

#### Glutamate and GABA vesicular transporters

Three isoforms of VGLUTs (VGLUT1, VGLUT2, and VGLUT3) underlie the vesicular uptake of glutamate in CNS neurons and thus represent markers of glutamatergic transmission. They share salient structural and functional characteristics but have differential cellular localization and functional properties, suggesting isoform-specific physiological roles (Fremeau et al., 2004; Chaudhry et al., 2008). In the CNS, VGLUT1, and VGLUT2 show complementary patterns of expression mainly in subset of glutamatergic neurons (Fremeau et al., 2001; Varoqui et al., 2002). VGLUT3, by contrast, is mostly expressed in neurons that were identified as non-glutamatergic. A broader examination of the distribution and functions of VGLUTs subsequently revealed that also VGLUT1 and VGLUT2 can be expressed in non-glutamatergic neurons, in consequence giving the strong indication that glutamate may very well act as a co-transmitter in many types of neurons. The presence of a VGLUT isoform in non-glutamatergic axon terminals indicates that glutamate can be either co-released by these axons or that it may contribute to an increased filling of synaptic vesicles with the primary transmitter, and consequently, increase the release of this transmitter. This has been demonstrated for cholinergic and serotonergic neurons, which contain VGLUT3 (Amilhon et al., 2010), and in dopaminergic neurons (Hnasko et al., 2010) and GABAergic neurons (Zander et al., 2010), which contain VGLUT2.

Only one vesicular transporter for GABA and glycine is known – the vesicular inhibitory amino acid transporter (VIAAT = VGAT; McIntire et al., 1997; Sagne et al., 1997). The transporter is co-expressed with markers for GABAergic and glycinergic synapses including GABA and glycine themselves, the GABA-synthesizing enzymes GAD65 and GAD67, the membrane glycine transporter GlyT2, and GABA membrane transporter (GAT1; Sagne et al., 1997; Chaudhry et al., 1998; Dalby, 2003). In summary, the co-expression of VGAT and the GAD enzymes determines the GABAergic phenotype (Wojcik et al., 2006), whereas, the co-expression of VGAT with the neuronal membrane glycine transporter GlyT2 is responsible for the glycinergic phenotype of a neuron (Gomeza et al., 2003).

#### Glutamate and GABA membrane transporters

The primary function assigned to the sodium-dependent membrane glutamate transporters (EAATs) is to maintain the extracellular glutamate concentration in the low micromolar range, allowing glutamate to be used as a signaling molecule in the brain and preventing its cytotoxic effects. Five high-affinity membrane glutamate transporters have been cloned from mammalian tissue: astrocyte-specific glutamate transporter (GLAST), glutamate transporter 1 (GLT1), excitatory amino acid carrier 1 (EAAC1), excitatory amino acid transporter 4 (EAAT4) and 5 (EAAT5). Immunocytochemical studies have revealed that GLAST and GLT1 are localized primarily in astrocytes (Shashidharan and Plaitakis, 1993; Tanaka et al., 1997), while EAAT3 and EAAT4 are distributed in neuronal membranes (Furuta et al., 1997).

GABA membrane transporter (GAT1) was the first of four GABA plasma membrane transporters isolated from rat brain (Radian et al., 1986; Guastella et al., 1990), which recognize GABA as a substrate (Dalby, 2003; Sarup et al., 2003). GAT1 ultrastructural localization is limited to axon terminals form symmetrical synapses (i.e., GABAergic) in the rat cerebral cortex but it can be also be expressed to some extent in proximal astrocytic processes, while the other transporters (GAT2-4) are located in astrocytic processes (Minelli et al., 1995; Ribak et al., 1996; Biedermann et al., 2002).

Transporters mediating reuptake of neurotransmitters have long been considered as selective glutamatergic and GABAergic neuronal markers. However, there is increasing evidence that neurons can co-express membrane transporters for various other transmitters. The key examples are the plasma membrane glutamate EAAT4 and EAAT3 transporter subtypes, which are localized on GABAergic Purkinje cells in the cerebellum (Rothstein et al., 1994; Furuta et al., 1997) and on hippocampal GABAergic neurons, respectively (Raiteri et al., 2002). Conversely, the plasma membrane GABA transporter GAT1 type co-exists with plasma membrane glutamate transporters on glutamatergic nerve terminals in spinal cord (Raiteri et al., 2005).

## NEUROTRANSMITTER PHENOTYPE MARKERS PRESENT IN THE GRANULE CELLS AND THEIR MOSSY FIBER

The hippocampal formation consists of GCs in the dentate gyrus and pyramidal cells in the CA1 and CA3 areas. The other type of neurons, the interneurons, are present in all hippocampal areas. The principal cells are interconnected by glutamatergic synapses, forming the “trisynaptic pathway” (Andersen et al., 1971; Storm-Mathisen, 1977). The GCs of the dentate gyrus receive excitatory glutamatergic input from layer II pyramidal cells of the entorhinal cortex (Steward and Scoville, 1976) and project to CA3 pyramidal cells. From there, they project to CA1 cells, which in turn project to the subiculum and back to the entorhinal cortex (Andersen et al., 1971; Amaral et al., 1990). The GCs show highly conserved properties across species (Seress and Frotscher, 1990) and are born continuously throughout life (Altman and Bayer, 1990), a feature that may be related to their role in memory formation (Schmidt-Hieber et al., 2004). There are approximately one million GCs within the rat dentate gyrus, all projecting thin unmyelinated axons into the stratum lucidum of the CA3, adjacent to the cell body layer. MFs form synaptic contacts with complex spines on proximal apical dendrites of CA3 pyramidal cells (Claiborne et al., 1986; Amaral et al., 1990) and various types of interneurones (Acsády et al., 1998).

Mossy fibers display three main types of presynaptic terminal exist along the entire length of the main axon: (1) large mossy boutons (4–10 mm in diameter), (2) filopodial extensions (0.5–2.0 mm) that emerge from the large mossy boutons, and (3) small *en-passant* varicosities (0.5–2.0 mm; Amaral, 1979; Frotscher, 1985; Claiborne et al., 1986; Acsády et al., 1998). The total number of large mossy boutons along a single axon is about 10 in the hilar region and about 12 (range 10–18) in the CA3 region, with the terminals being distributed approximately every 150 mm along the MF axon (Acsády et al., 1998). The large MF terminals, which contain up to 35 individual release sites envelop postsynaptic complex spines (“thorny excrescences”) found on the proximal apical dendrite of CA3 pyramidal cells. A single CA3 pyramidal neuron typically receives input from about 50 MF boutons (Amaral et al., 1990). In the hilus, the so called “mossy cells” constitute the principal target of the large mossy terminals. Small filopodial extensions and *en passant* terminals found on the parent axon predominantly innervate inhibitory interneurons in both the hilus and stratum lucidum (Acsády et al., 1998). These terminals form single, often perforated, asymmetric synapses on the cell bodies, dendrites, and spines of GABAergic inhibitory interneurons (McBain, 2008). Although large MF boutons do not typically innervate GABAergic interneurons, they have occasionally been observed to directly contact dendrites interneurons in the guinea pig (Frotscher, 1985). These MF-interneuron synapses recruit feed-forward inhibition onto the CA3 pyramidal cells. Thus, the morphological properties of the MF synapse are consistent with the view that it forms a powerful, but sparse synaptic connection (Rollenhagen and Lübke, 2010).

### GLUTAMATE

The primary excitatory neurotransmitter released from MF terminals is glutamate (Crawford and Connor, 1973; Storm-Mathisen, 1981; Storm-Mathisen et al., 1983). MFs-CA3 pyramidal cell synapses are one of the most powerful glutamatergic input, “detonator” synapses (Urban et al., 2001; Henze et al., 2002; Mori et al., 2007). However, the high strength requires some winding up, in the form of frequency dependent facilitation (Henze et al., 2002; Mori et al., 2007) and it is coupled with a very low basal firing rate of GCs *in vivo* (<0.5 Hz). In fact, many GCs appear not to fire action potentials for extended periods of time (Jung and McNaughton, 1993).

### GABA AND GAD

Sandler and Smith (1991) found GABA contained in MF terminals that made asymmetric synaptic contact with spines arising from large dendrites of CA3 pyramidal cells. GC bodies, however, did not show GABA immunoreactivity. They also showed the colocalization of GABA and glutamate within the same terminals with electron microscopy. Later, Sloviter et al. (1996) conclusively demonstrated that GABA and its synthesizing enzyme, GAD67, co-existed in the MFs of rats, monkeys, and humans. These authors additionally demonstrated that seizures upregulated the contents of both isoforms of GAD and GABA (Sloviter et al., 1996). Other authors showed that GAD67 and its mRNA (but not GAD65) were transiently upregulated after seizures provoked by kainic acid or by the kindling method in the rat (Schwarzer and Sperk, 1995; Lehmann et al., 1996; Ramírez and Gutiérrez, 2001; Maqueda et al., 2003). Quantitative immunogold analysis confirmed the co-existence of these amino acids within single MF terminals, and in close relationship with synaptic vesicles, where GABA concentration was found to be lower than that of glutamate (1.5 vs. 7.2 mM, respectively; Bergersen et al., 2003). Recently, Sperk et al. (2012) showed a marked enhancement of the expression of one of the two key enzymes of GABA synthesis – GAD67 – in MFs of epilepsy patients.

Therefore, if the GCs have the necessary enzyme for the synthesis of GABA and GABA itself, the GCs indeed synthesize GABA that could probably be used as a neurotransmitter.

### VESICULAR GLUTAMATE TRANSPORTER (VGLUT1 AND VGLUT2) AND VESICULAR GABA TRANSPORTER (VGAT)

Bellocchio et al. (1998) and Takamori et al. (2000) conclusively demonstrated that a protein, previously identified as brain specific Na^+^-dependent phosphate transporter (BNPI; Ni et al., 1994) – now known as VGLUT – endows the cells with a functional glutamatergic phenotype. Moreover, these authors showed that the protein was both in the DG and MF terminals. On the other hand, the first evidence of the presence of VGAT mRNA in MF was obtained from an enriched MF synaptosome preparation and from micro slices of the DG, where the possibility of having GABAergic cells could not be ruled out (Lamas et al., 2001). However, in that work, the expression pattern of the mRNA coincided with the upregulation of GAD and appearance of GABA-mediated transmission on MF stimulation (see below). Soon after, with the use of single-cell PCR, Gómez-Lira et al. (2005) conclusively demonstrated that mRNA was contained in the GCs and that it was expressed in a developmental- and activity-dependent manner. Despite the presence of VGAT mRNA, some immunohistological studies failed to detect the VGAT protein in the MFs (Chaudhry et al., 1998; Sperk et al., 2003). Bergersen et al. (2003, 2008), by using an electron microscopic immunogold method, could show that GABA was present together with glutamate in all large MF terminals and that both amino acids were associated with synaptic vesicles.

**FIGURE 1:**
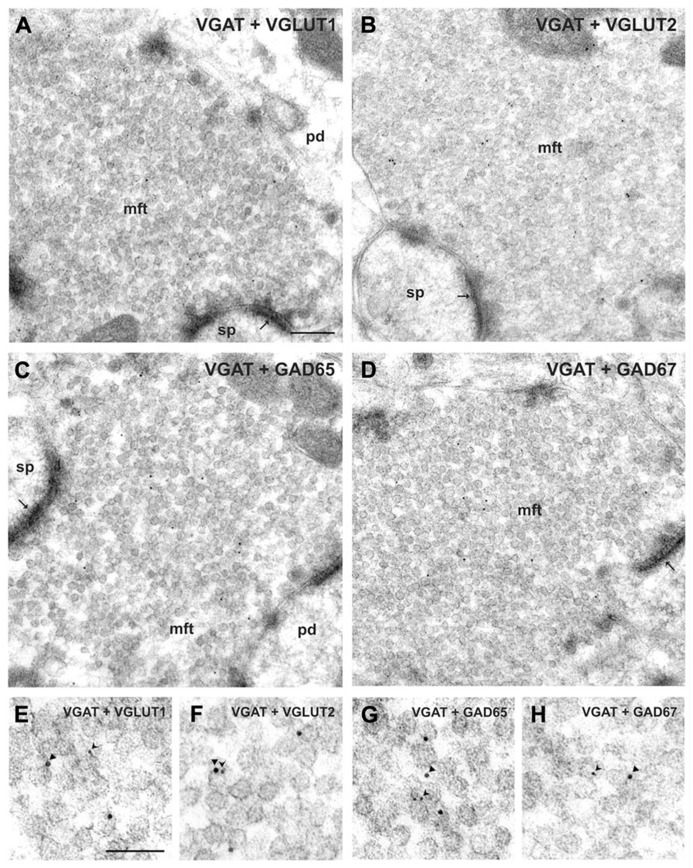
**Co-localization of VGAT with either GAD or VGLUT isoforms in hippocampal mossy fiber terminals. (A,B)** Mossy fiber terminals (mft) of the CA3 area were double-immunolabeled with the guinea pig antiserum against VGAT (10 nm gold particles, arrowheads in **E**, **F**) and rabbit antisera against either VGLUT1 and VGLUT2 (5 nm gold particles, forked arrowheads in **E**, **F**, respectively). Note the SV exhibiting putatively co-localized immunogold signals for VGAT and VGLUT2 shown in **(F)**. **(C,D)** Mossy fiber terminals were double-immunolabeled with the guinea pig antiserum against VGAT (10 nm gold particles, arrowheads in **G**, **H**) and rabbit antisera against either GAD65 or GAD67 (5 nm gold particles each, forked arrowheads in **G**, **H)**, respectively. **(E–H)** Represents details at higher magnification. pd, pyramidal dendrite; sp, spine; thin arrows indicate asymmetric contacts. Bars given represent: **(A–D)**, 200 nm; **(E–H)**, 100 nm (from Zander et al., 2010).

In a recent work, the presynaptic co-localization of VGLUTs and VGAT proteins in glutamatergic hippocampal MF terminals was conclusively demonstrated (Zander et al., 2010). We found that the GABA transmitter phenotype is preserved in the mature brain as indicated by the presence of GABA synthesizing enzymes GAD65 and GAD67, as well as the VGAT (**Figure 1**). Also, unique co-existence of VGAT and VGLUT was observed in cerebellar MF terminals by absence of VGAT immunoreactivity in other glutamatergic cerebellar parallel fiber (VGLUT1-positive) and climbing fiber (VGLUT2-positive) terminals. These findings provide strong morphological evidence for synaptic co-existence of glutamatergic and GABAergic transporters in adult glutamatergic MF terminals. Furthermore, we carried out postembedding immunogold electron microscopy with vesicular immunoisolation experiments in order to determine vesicular co-localization VGAT and VGLUT in the MF synapse. The immunoisolation experiments from whole brain of adult rats further confirmed that VGLUT1 immunoisolates also contain VGLUT2 and, conversely, VGLUT2 immunoisolates show VGLUT1 immunoreactivity, indicating the vesicular co-localization of both VGLUTs. VGLUT2 immunoisolates harbor, in addition, VGAT and VGAT immunoisolates also contain VGLUT2. Transporter-specific immunoisolations from rat brain at different postnatal stages (P5 and P15) showed a pronounced vesicular co-localization of VGLUT2 and VGAT during these early developmental stages. Transmitter uptake studies using Glu and GABA proved the functional integrity of immunoisolated vesicles. In fact, VGAT immunoisolates were found to concentrate Glu in addition to GABA. Using the specific inhibitor trypan blue we found that VGLUT activity improves GABA, as well as monoamine uptake into synaptic vesicles. Thus, VGLUT activity modulates transmitter storage in a substrate-independent manner. Using immunogold detection and electron microscopy, Zander et al. (2010) proposed that glutamatergic MF terminals contain vesicles co-expressing either VGLUT1 or VGLUT2 and VGAT. This confirms that both vesicle transporters are in the same terminals, which strongly support the hypothesis of co-release of glutamate and GABA. However, direct proof of co-existence of vesicular transporters of different kind in single vesicles cannot be provided by the limitations of the immunogold technique. Although it is possible that both transporters can be expressed by single vesicles, electrophysiological experiments suggest that these vesicular transporters could be sorted to different pools of vesicles (Walker et al., 2001; Beltrán and Gutiérrez, 2012; see, Activation of the MFs Normally Produces Gabaergic Responses During Development).

## GABA MEMBRANE TRANSPORTER

It was found that MF terminals from control and epileptic animals capture GABA and nipecotic acid blocks this uptake (Gómez-Lira et al., 2002), suggesting that they normally express GAT-1, further indicating that these cells are actually potentially GABAergic. This has also been confirmed by immunohistological techniques (Frahm et al., 2000; Sperk et al., 2003). These data show another GABAergic marker in the MFs, which allows them to take GABA from the extracellular millieu into the terminals. Its presence, however, is not what determines that MFs release GABA, moreover, blockage of this transporter enhances MF-evoked GABA responses (Vivar and Gutiérrez, 2005).

### GABAA RECEPTORS

For a neurotransmitter to exert its action, the corresponding receptor(s) should be in the subsynaptic site. In other words, pyramidal cells of CA3 and interneurons in the hilus and CA3 should have GABAA receptors together with glutamate receptors apposed MF terminals. Bergersen et al. (2003) demonstrated that GABAA receptors coexist with AMPA type glutamate receptors apposed MF terminals using immunogold detection. Moreover, they also detected presynaptic GABAA receptors in the MFs themselves (Bergersen et al., 2003; Ruiz et al., 2003), which can be activated by GABA released from interneurons or even from the MFs themselves (Treviño and Gutiérrez, 2005; Alle and Geiger, 2007), and which can exert strong modulation of MF excitability (Ruiz et al., 2010). Thus, GABA released by the MFs can activate post-synaptic and presynaptic GABAA and B receptors to produce collateral potentiation or inhibition (Ruiz et al., 2003, 2010; Treviño and Gutiérrez, 2005; Cabezas et al., 2012).

### PHYSIOLOGICAL AND PHARMACOLOGICAL MARKERS

Because MFs neurotransmission is usually studied in the slice preparation, and activation of fibers other than MFs is always a possibility, there is an agreement on three principal characteristics that glutamatergic, and thus GABAergic, synaptic responses of putative MF origin should have to be recognized as true MF responses: (1) strong frequency-dependent potentiation (>300%) in response to modest increases in stimulation frequency; (2) robust NMDA-independent LTP; and (3) depression (>80%) of the responses by activation of mGluR, which are present in the MFs. These and several other physiological characteristics of MF transmission and its plasticity are thoroughly reviewed elsewhere (Henze et al., 2000; Lawrence and McBain, 2003; Nicoll and Schmitz, 2005; Jaffe and Gutiérrez, 2007; Galván et al., 2011; Ruiz and Kullmann, 2013). Moreover, these characteristics are preserved in cultured GCs establishing autapses (Rost et al., 2010) or in synapses made by GCs co-cultured with pyramidal cells and interneurons of CA3 (Osorio et al., 2013).

## DOES THE PRESENCE OF ALL THE GABAERGIC MARKERS IN THE GRANULE CELLS AND THEIR MFS MAKE THEM GABAERGIC AND INHIBITORY?

The presence of markers of the GABAergic phenotype in the GCs should not necessarily make them GABAergic and inhibitory. However, evidence has been accumulating in favor of them expressing, besides a glutamatergic phenotype, a GABAergic phenotype, with inhibitory functions. We will make a comprehensive account of what we now know about the GABAergic signaling of the MFs to be able to orderly attempt to speculate on the outcome of all of the possibilities and their interactions (Mody, 2002).

The first electrophysiological evidence of GABAergic transmission from the MFs to CA3 was in total agreement with the observations that seizures transiently upregulated the expression of the GABAergic markers GAD65 and GAD67 (Schwarzer and Sperk, 1995; Lehmann et al., 1996; Sloviter et al., 1996; Ramírez and Gutiérrez, 2001) and VGAT (Lamas et al., 2001). In accordance with this evidence, Gutiérrez and Heinemann (1997, 2001) and Gutiérrez (2000) found monosynaptic GABA-mediated transmission from the DG to CA3 in kindled epileptic but not in control healthy rats, i.e., activation of the MFs in the presence of glutamatergic blockers evoked fast, m-GluR-sensitive IPSPs with the same latency as the EPSP evoked before blocking glutamatergic transmission, and blocked by bicuculline. By contrast, perfusion of glutamatergic antagonists to slices of healthy animals prevented all synaptic responses, as expected for blockade of monosynaptic, glutamatergic and thus, polysynaptic, GABAergic inhibition. Another group provided independent evidence that strongly supported the hypothesis that MFs corelease glutamate and GABA. In contrast to the aforementioned data, Walker et al. (2001) showed that in non-epileptic, young guinea pigs, minimal stimulation of the MF in the presence of glutamatergic antagonists provoked monosynaptic GABAergic responses with the pharmacological and physiological profile of transmission of MF origin. Because all control animals used in the former experiments were adult Wistar rats (Gutiérrez, 2000), a possible source of discrepancy with the results obtained by Walker et al. (2001) could be attributed to the developmental stage or to the different species used. However, the discrepancy does not seem to arise from the use of different species because there is evidence of the presence of GABA and GAD in the MFs of the rat, guinea pig, and even in human and non-human primates (Sandler and Smith, 1991; Sloviter et al., 1996; Bergersen et al., 2003; Sperk et al., 2012).

### ACTIVATION OF THE MFS NORMALLY PRODUCES GABAERGIC RESPONSES DURING DEVELOPMENT

The aforementioned data suggested the possibility that MFs of young rodents may naturally release GABA while adult animals lose this capability. We recorded responses of CA3 pyramidal cells to MF stimulation throughout development in Wistar rats (Gutiérrez et al., 2003) and found that in the developing rat MF stimulation in the presence of glutamatergic blockers normally evoked a monosynaptic GABA-mediated response, which was depressed by activation of mGluR and completely blocked by bicuculline. This response was depolarizing during the first week of life (6 days old), turned to hyperpolarizing from the second week (10 days old) and, from day 21, the number of cells having the MF-GABAergic response declined rapidly until they were no longer detected by day 23–24 (Gutiérrez, 2003).

**FIGURE 2:**
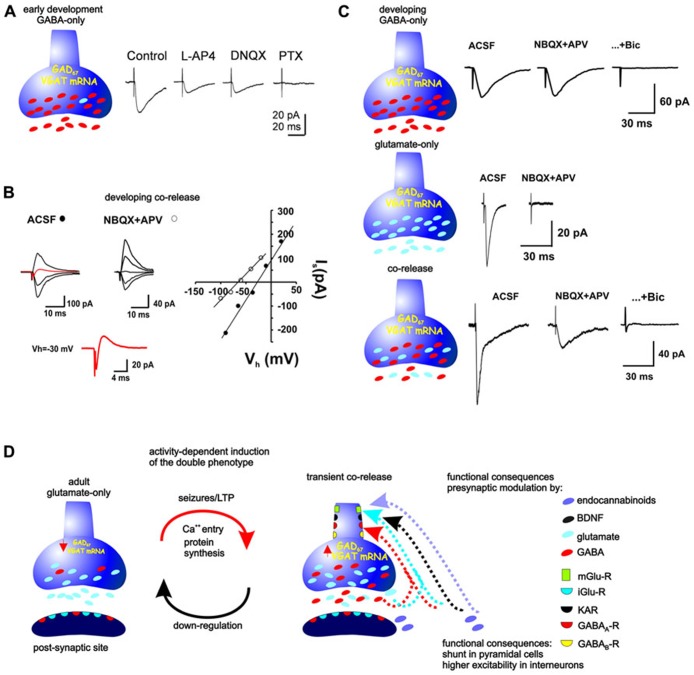
**Neurotransmitter phenotype of the MFs during development and its plasticity in the adult. (A)** During the first 5 days of life, MFs are GABAergic. Minimal stimulation provokes synaptic currents that are partially inhibited by the glutamatergic antagonist DNQX and completely blocked by the GABAA-R antagonist, picrotoxin (modified from Safiulina et al., 2006). **(B)** From postnatal day 5–6 and up to the end of postnatal day 21–22, the synaptic responses evoked by stimulation of MF boutons can consist of inward and outward currents, consistent with co-release of glutamate and GABA (red traces at Vh = 30 mV), whereby their reversal potential is much more positive than that expected for a GABA-R-mediated current or much more negative than that expected for a glutamate-R-mediated current. Block of glutamatergic receptors isolates GABAergic responses, shifting the reversal potential to a more negative value (modified from Beltrán and Gutiérrez, 2012). **(C)** Other responses observed by stimulation of single, identified MF boutons during this period consist of glutamatergic-only or GABAergic-only currents (modified from Beltrán and Gutiérrez, 2012). **(D)** Stimulation of MFs in adult preparations provoke glutamate-R-mediated responses, however, seizures or repeated LTP-like stimulation up-regulate the GABAergic markers and MF activation provokes monosynaptic GABAR-mediated responses in their target cells. The release of glutamate and GABA from the MFs can serve presynaptic modulation of parallel MFs or of the releasing fibers themselves. Mossy fibers as well as the post-synaptic sites contain glutamate and GABA receptors. Moreover, the MFs themselves express these and receptors to BDNF, endocannabinoids, kainite, and metabotropic glutamate and GABAreceptors, which have been shown to modulate both glutamate and GABA release.

These data, together with those of Walker et al. (2001) using the young guinea pig, established that MF GABA release was a characteristic of the developing rodent. Moreover, their thorough analysis of the physiological (frequency potentiation, LTP) and pharmacological (mGluR sensitivity) characteristics indicated that the GABAergic monosynaptic responses have indeed their origin in the MFs. Additionally, they suggested that glutamate and GABA should be released from different vesicles because the synaptic events were produced by either activation of glutamate receptors, GABA receptors, or both (Walker et al., 2001; Beltrán and Gutiérrez, 2012). Were both amino acids co-released from the same vesicles, each synaptic event would contain both components. Finally, these characteristics of MF transmission were also corroborated and extended by the group of Cherubini, which provided further evidence for a GABAergic phenotype of the MFs at even earlier ages. They suggested that MF transmission is primarily GABAergic during the first posnatal days, at P0–P6 (Safiulina et al., 2006). From P6, the co-expression of the glutamatergic and GABAergic phenotypes in a single pathway provides an efficient and rapid synergism on their target cells during development (Gutiérrez et al., 2003; Kasyanov et al., 2004). Indeed, it was shown that “pairing” spontaneous GABA-mediated giant depolarizing potentials with MF stimulation, which produces GABAA receptor-mediated synaptic responses, induces a persistent increase in synaptic efficacy at MF–CA3 synapses and proposed that different fibers or boutons release either glutamate or GABA or both (Kasyanov et al., 2004), which has been recently directly proven (Beltrán and Gutiérrez, 2012). The sequence of the development of the initially GABAergic-only, dual GABAergic-glutamatergic, and finally glutamatergic-only phenotype of the MFs is summarized in **Figure 2**.

Additionally, as in the case of glutamatergic signaling, the MF-GABAergic signaling is presynaptically tightly controlled by GABA, kainate and CB1 receptors (Safiulina et al., 2010). Indeed, CB1 receptors, which are expressed in the GCs at very young ages, can be activated by endocannabinoids released by the postsynaptic cells, thus, counteracting enhanced excitability induced by the excitatory action of GABA at that developmental stage (Caiati et al., 2012). It is interesting that activation of type III mGluRs have a much stronger inhibitory effect than that of type II MGluRs on MF GABA responses evoked both in the guinea pig and rat (Walker et al., 2001; Gutiérrez, 2003; Kasyanov et al., 2004) and the glutamate present in the extracellular milieu reaches concentrations that can also strongly inhibit MF release by acting on GluK1 receptors (Caiati et al., 2010; **Figure 2**). Finally, MF-GABA release itself controls subsequent release through activation of GABAB receptors, increasing the number of silent synapses (Safiulina and Cherubini, 2009). Recently, Cabezas et al. (2012) have shown that GAD67-containing MFs of juvenile mice can release GABA to modulate MF excitability by acting on presynaptic GABAB receptors, and suggest that their primary function is instructing the pre-synaptic element. Interestingly, despite detecting the release of GABA from the MF, they could not record GABAA-mediated responses in post-synaptic cells.

GABAA receptors appear before AMPA receptors and GABA provides the necessary depolarization needed to activate CA3 pyramidal cells. During the first postnatal week, GABA has a depolarizing action, as observed by somatic recordings, and has been proposed to exert a trophic effect during development (Ben-Ari et al., 1994, 1997). From the second week of age, GABA exerts hyperpolarizing actions, so what would be the function of MF-GABA after that period and up to the third week of life? Interestingly, it was shown that although GABA actions are hyperpolarizing from the second week, as measured in the soma, MF-GABA still produces a depolarizing actions in the pyramidal cell dendrites until the end of the third week (Romo-Parra et al., 2008). This is explained by a relocation of the NKCC transporter from the somatic to the dendritic region at that developmental stage (Marty et al., 2002). Thus, GABA release from the MFs may contribute, both pre- and postsynaptically, to the refinement of neuronal connectivity before the establishment of the adult neuronal circuit (**Figure 2**).

### ACTIVATION OF THE MF IN THE ADULT TRANSIENTLY PRODUCES MIXED GLUTAMATERGIC-GABAERGIC RESPONSES AFTER A PERIOD OF ENHANCED EXCITABILITY

In slices from mature control rats, the perfusion of glutamatergic antagonists blocked all synaptic responses in CA3 to DG stimulation. However, in slices from kindled rats, the perfusion of glutamatergic antagonists blocked the EPSP but a fast monosynaptic inhibitory postsynaptic potential (IPSPs) remained (Gutiérrez and Heinemann, 1997, 2001; Gutiérrez, 2000). This GABAA receptor-mediated response had the same latency as the EPSP obtained previous to its blockage and remained in low Ca^2^^+^ conditions without modifying its latency. As expected of responses of MF origin, activation of mGluR depresses these monosynaptic GABA responses (Gutiérrez, 2000; Gutiérrez and Heinemann, 2001). Importantly, the presence of these responses was transient because they are not present if the same type of recording is conducted 1 month after the last of several kindled seizures. On the other hand, it was established that the MF-GABAergic responses could be observed after producing a single seizure, which does not constitute a permanent epileptic state. Thus, these data together show that it is not the preexistence of an epileptic condition, nor the possible sprouting of interneurons during its evolution that explains why pyramidal cells responded with a GABAergic response on MF stimulation. Furthermore, the induction of the expression of MF GABA-mediated transmission can also occur after LTP-like stimulation *in vitro* of the perforant path, which demonstrated that this is a presynaptic phenomenon. Moreover, providing the LTP-like stimulation directly over the GCs during blockade of synaptic potentials (with NBQX and APV) also induced the MF GABAergic responses. This evidence rules out activation of interneurons as origin of mGluR-sensitive, GABAergic responses evoked by DG stimulation. Finally, the induction of MF-GABA transmission was shown to depend on protein synthesis (Gutiérrez, 2002; Romo-Parra et al., 2003; **Figure 2**).

As might be expected, the major target cells of the MFs, the interneurons in area CA3 (Acsády et al., 1998), also receive this dual input (Romo-Parra et al., 2003; Safiulina et al., 2006), as do the mossy cells in the hilar region (Bergersen et al., 2003). Whether there are different types of modulation of the MF GABAergic input onto each type of GABAergic interneuron of CA3 is still unknown, however, current data in our laboratory indicates that this is the case. This can be expected given the evidence of compartmentalization of MF actions on different types of target cells (MacCaferri et al., 1998; Galván et al., 2011).

### THE GABAERGIC MARKERS ARE TRANSIENTLY UP-REGULATED WHEN GABAERGIC TRANSMISSION FROM THE MFS IS TRANSIENTLY EXPRESSED

As previously noted, the appearance of the GABAA-receptor-mediated monosynaptic responses after kindled or PTZ-induced seizures is transient and coincides with the transient upregulation of GAD (Schwarzer and Sperk, 1995; Sloviter et al., 1996; Ramírez and Gutiérrez, 2001), GABA (Gómez-Lira et al., 2002), and VGAT mRNA (Gómez-Lira et al., 2005). We know that prevention of protein synthesis prevents the MF GABAergic responses to appear, but at this time we do not know which protein is the limiting one for the ultimate release of GABA from the MFs (**Figure 2**). It is interesting that MF boutons in the adult contain detectable levels of GABA and GAD (Sloviter et al., 1996) and still, GABA does not seem to be released in the rat after 22 days of age, unless a period of hyperexcitability is induced.

Other epilepsy models have also shown that GAD and/or GABA are upregulated after seizures (Sloviter et al., 1996; Sirvanci et al., 2005). Finally, although MF GABA synaptic transmission has not been corroborated in human epileptic tissue, the expression of GAD and GABA in epileptic MFs is enhanced as compared to post mortem controls, suggesting the emergence of a compensatory, protecting mechanism against further excitability (Sperk et al., 2012).

### FUNCTIONAL RELEVANCE OF THE DUAL GLUTAMATERGIC-GABAERGIC PHENOTPYE OF THE MOSSY FIBERS

We have discussed some of the possible implications of glutamate-GABA corelease from the MFs during development (see activation of the MFs normally produces GABAergic responses during development), most of which have been confirmed by several research groups. The emergence of a GABAergic phenotype in adult, glutamatergic GCs has also been extensively studied. As we have already mentioned, it is not only the presence of the markers of the GABAergic phenotype that makes them GABAergic and inhibitory. A wealth of evidence supports the assumption that, in the adult, MFs actually become inhibitory when they express GABAergic markers. Because MFs release GABA onto both, pyramidal cells and interneurons (Romo-Parra et al., 2003), the overall effect of MF input on CA3 could be disinhibitory, which has been discarded. Moreover, activation of the MF in hippocampal slices from epileptic, but not from control animals, produced GABA-mediated field responses in Stratum lucidum (SL) of CA3, which could be pharmacologically isolated (Treviño and Gutiérrez, 2005) and that were inhibited by mGluR activation and sensitive to change in stimulation frequency. Thus, the MF pathway functions as a GABAergic projection in the hippocampus that can alter CA3 population activity after seizures. Moreover, this GABAergic transmission from the MFs activates presynaptic GABAA receptors located in other MF axons (MFA), producing collateral inhibition. The activation of the DG after applying a kindling-like protocol *in vitro*, or after producing one or several seizures *in vivo*, results in a graded and reversible increase of inhibitory conductances in pyramidal cells, while in Sl-interneurons, an increase of excitatory conductances occurs. Thus, interneurons reach more depolarized membrane potentials on DG activation yielding a high excitatory postsynaptic potential-spike coupling, while the contrary occurs in pyramidal cells. This effective activation of feedforward inhibition is synergized by the emergence of direct DG-mediated inhibition on pyramidal cells. These factors force the synaptic conductance to peak at a potential value close to resting membrane potential, thus producing shunt inhibition and decreasing the responsiveness of CA3 output cells to MF input (Treviño et al., 2011). Therefore, the time-locked interaction of the excitatory and inhibitory conductances in pyramidal cells tends to inhibit spiking activity and may produce a shunting effect of other excitatory inputs (see also Monier et al., 2008). Thus, the contribution of this GABAergic input is consistent with the hypothesis that shunt inhibition restricts the firing of action potentials, even during a partial build-up of depolarization. On the other hand, spontaneous oscillations of CA3 were analyzed in control and epileptic animals and we found that spontaneous beta-gamma oscillations of this region are tonically suppressed by the MF input and the activation of mGluR relieves this tonic inhibition. By contrast, oscillations observed in slices from control animals were not altered. A second evidence for the MF GABAergic modulation of CA3 oscillatory activity was the lack of effect of inhibition when the MF tract was disrupted (Treviño et al., 2007).

Thus, the emergence of co-release of glutamate and GABA from the MFs in the epileptic brain can reflect the put in play of excitation-restraining, antiepileptic, and neuroprotective actions in response to extreme activity conditions. It has been proposed that the DG serves as a filter (Heinemann et al., 1992) limiting the transfer of information from the entorhinal cortex to the pyramidal layer of the hippocampus. In chronic temporal lobe epilepsy and respective animal models, MF rearrange such that they innervate also neighboring glutamatergic projection cells. Co-release of GABA and glutamate after seizures will thus prevent activation of neighboring cells and may also prevent activation of mossy cells. Reduced activation in these cells may also have impact on the reduced seizure susceptibility which is frequently observed after temporal lobe seizures.

### NEWBORN GRANULE CELLS IN THE ADULT CAN REGULATE THE EXPRESSION OF GABAERGIC MARKERS

Because GCs are generated throughout life, and GCs normally express GABA markers during their development, it was reasonable to propose that the newborn neurons in the adult rodent recapitulate the development of the neurotransmitter phenotype of GCs generated during embryonic and early postnatal development. Interestingly, single seizures produce a strong up-regulation of GAD67 in cells located in the infragranular layer of the DG, where most of GCs born in the adult are located (Ramírez and Gutiérrez, 2001), which suggested that GCs born in the adult can express a GABAergic phenotype. Toni et al. (2008) explored this possibility by stimulating MF boutons (light evoked transmitter release) and electrophysiologically recording in the postsynaptic cells. Their analysis, however, was conducted in cells labeled 4 months prior to the experiments, which time is probably beyond the transient period when they express GABAergic markers. If the GABAergic phenotype is transiently expressed, then its markers should be present at younger ages after retroviral infection. We determined that 15–20% of developing GFP^+^ GCs expressed GAD67/GABA, while the adult, GFP^+^ GCs did not express these markers at 30 days post injection. However, while these results refer to the somatic expression of the GABAergic markers, we observed that GFP^+^ MF boutons expressed GAD67 at all ages (Lara et al., 2012). There may be several possible reasons why only this percentage of developing GFP^+^ cells expresses GAD67, while adult GFP^+^ cells do not. Indeed, it has been shown that many, but not all GCs born perinatally express the GABAergic markers in their somata (Gutiérrez et al., 2003; Maqueda et al., 2003). Also, GCs are born continuously but different cohorts of newborn can be found at a given time (Mathews et al., 2010; Piatti et al., 2011). It is possible that some cohorts do express GAD67 (as found in developing rats; Gutiérrez et al., 2003) and they may be differentially integrated to circuits. Noteworthy, while GFP^+^ GCs somata may not express GAD67/GABA, we found that their MFs did express GAD67, as has been shown to occur normally in developing and adult MFs (Lara et al., 2012). A specific GAD67-expressing population of newborn cells may be distributed into distinctive networks to serve a function in cognitive tasks. Thus, the possible existence of reserve pools of neurons that can respecify their neurotransmitter phenotype (Dulcis and Spitzer, 2011), could apply to newborn GCs that can be recruited into dynamic, existing circuits. Interestingly, newborn GCs increase after seizures (Parent et al., 1997) and it has been shown that they overexpress GAD67 (Jiang et al., 2004). Additionally, newborn GCs in culture conditions can express a GABAergic phenotype in a manner dependent on activity, and by kainate or BDNF exposure (Babu et al., 2007), as previously described to occur in cultured adult GCs (Gómez-Lira et al., 2005).

Together, this information suggests that GCs born in the adult may release GABA just before their complete incorporation to the circuitry, probably to act as a trophic factor or to instruct the formation of new synapses. This possibility is supported by the fact that stimulation of the DG in slices of adult rats do not normally evoke synaptic responses in CA3 in the presence of glutamatergic antagonists, though they continue to produce new neurons. On the other hand, applying LTP stimulation over the DG in the presence of glutamatergic antagonists, which does not initially evoke synaptic responses, originates the appearance of GABA-mediated responses in CA3 cells after its repetition (12 times) in a protein synthesis-dependent manner (Gutiérrez, 2002). This excludes the newborn GCs as the source of monosynaptic GABAergic responses. A caveat of these experiments, however, is that no paired recordings were conducted, so short range projections of newborn GCs could not be probed in a selective manner. Future experiments will determine whether the expression of GABAergic markers confer the GCs a true GABAergic phenotype.

## EVIDENCE AGAINST AND DIRECT EVIDENCE IN FAVOR OF THE GABAERGIC PHENOTYPE OF THE MFs

The wealth of data here presented strongly suggests that GCs are able to synthesize, vesiculate, and release GABA and glutamate on to their target cells during development and, in the adult GCs, after a period of enhanced excitability. Moreover, the physiological changes observed during the expression of the GABAergic phenotype are consistent with them being also inhibitory. Despite this, some works have appeared with interpretations for the “MF GABA responses” other than co-release. Some of these works do not find evidence of co-release rather than providing evidence against the hypothesis of co-release. Indeed, some reports fail to observe a given GABAergic marker, or a given characteristic of a “true” MF GABAergic response, usually because different experimental conditions have been used.

The first obstacle in accepting that GABA could be released from MFs was the apparent lack of its vesicular transporter, VGAT. Several authors failed to find the protein in the MFs (Sperk et al., 2003; Boulland and Chaudhry, 2012) and our initial results, although showing the presence of VGAT mRNA in MFs synaptosomes and DG, lacked the specificity so as to ascertain that VGAT was indeed present in GCs (Lamas et al., 2001). We later demonstrated with the use of single-cell PCR that its mRNA was indeed present, that its expression was modulated by age and activity in the adult (Gómez-Lira et al., 2005). Recently, using postembedding immunogold electronmicroscopy, we could reveal the co-localization of VGAT protein with VGLUTs in single MF boutons and the evidence also suggested that they were also in single vesicles (Zander et al., 2010). It has also been suggested that an unidentified GABA transporter may exist, also supported by the evidence that some GABAergic interneurons lack VGAT (Chaudhry et al., 1998; Boulland and Chaudhry, 2012). Finally, the diverging data may be due to technical differences. As derived from this, a direct demonstration of VGAT-dependent release from the MFs is still needed.

Mori et al. (2004) conducted paired recordings of connected GCs and pyramidal cells in cultured slices and did not find GABA-mediated responses in pyramidal cells on activation of GCs. Their conclusion was that the GCs do not release of GABA. We should remember, however, that these experiments were conducted under culture conditions with cells of more than 4 weeks of age. This does not constitute evidence against co-release because the experiments did not meet the conditions where co-release has been determined to occur: during development or in adult GCs, after a period of enhanced excitability. The same holds true for another report by Cabezas et al. (2012), who did not detect GABA-mediated postsynaptic currents in pyramidal cells by activation of coupled GCs in organotypic cultures. Interestingly, they did show that MFs released GABA that can act on GABAB receptors present in the presynaptic terminals, suggesting that GAD expression does not endow the MFs with a GABAergic phenotype, rather GABA’s primary function is to instruct the presynaptic element. Cultured slices have the inconvenience that the developmental stage of the cells is difficult to define and both, their excitability conditions and synaptic organization can be strongly affected by the culture method (Gutiérrez and Heinemann, 1999; De Simoni et al., 2003).

Uchigashima et al. (2007) questioned the putative MF origin of the GABAergic responses observed in CA3 on MF stimulation and suggested that they may be brought about, possibly, by stimulation of MF-associated interneurons (Vida and Frotscher, 2000; Losonczy et al., 2004). However, some of the responses they found were mildly sensitive to the activation of type II mGluR with DCG-IV (Uchigashima et al., 2007), while other monosynaptic GABAergic responses that they did observe to be more sensitive to mGluR activation were those in which L-AP4, a type III mGluR agonist, was used. Thus, they leave open the possibility that L-AP4 is indeed a better modulator of MF GABAergic transmission, albeit with reservations or without testing whether this was indeed the case. L-AP4 has been repeatedly shown to be strong inhibitor of MF GABA responses in the rat in comparison to DCG-IV (Gutiérrez, 2002; see also Safiulina et al., 2006; Treviño et al., 2007). Uchigashima et al. (2007) acknowledge that the GABAergic machinery, GAD and GABA, is present in the MF, although they did not detect VGAT (as already mentioned, this protein was, until recently, very difficult to detect with immunohistochemical methods, but postembedding electron microscopy has revealed its presence; Zander et al., 2010). On the other hand, the recognition of L-AP4-sensitive monosynaptic GABAergic responses as not of MF origin may be a false negative interpretation. Indeed, the number of boutons that release GABA or that co-release glutamate and GABA is low (Beltrán and Gutiérrez, 2012). Identical experiments should be done, as the work of Uchigashima et al. (2007) attempted, but without discarding possible positive results without proper controls (for example, there is evidence that L-AP4 is more effective than DCG-IV in inhibiting putative MF-GABA transmission and that L-AP4 does not affect IPSCs evoked by SL interneurons, which were not tested). Although at first sight, Uchigashima et al. (2007) provide evidence that is in contrast to what several groups have shown, there are some methodological differences in their work with respect to others that do not permit to state that they have found evidence against the release of GABA from the MF.

To avoid ascribing synaptic transmission to fibers other than MFs, an excitatory or an inhibitory response should be evoked on a pyramidal cell by an action potential of an identified GC. This task is unlikely to be accomplished using paired recordings on slices. Selective stimulation of MF boutons and recording from a post-synaptic cell in a slice is also a possibility to solve this problem, but because of the difficulty in finding the post-synaptic cell that responds to the bouton being stimulated and because only a number of boutons seem to co-release the amino acids, this second possibility can also be time-consuming and not ideal for these goals (Bischofberger et al., 2006).

**FIGURE 3:**
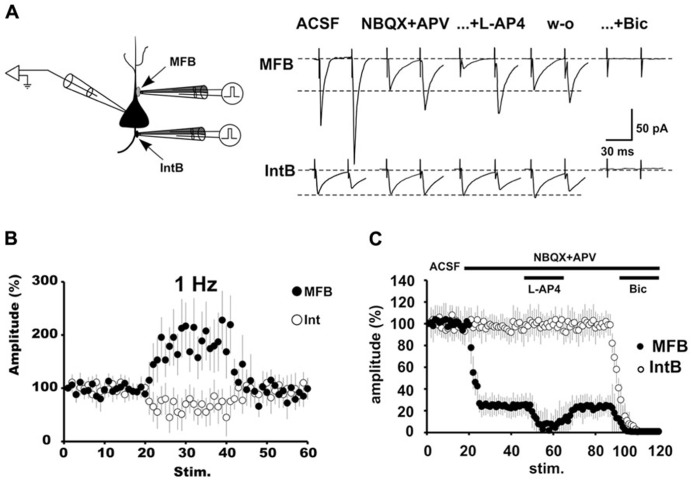
**The pharmacologically isolated GABAergic synaptic currents evoked by stimulation of MFBs have the signature of transmission of MF origin. (A)** Schematic depiction of an isolated pyramidal cell with a fluorescent MF bouton on the apical dendrite and an interneuronal bouton on the basal dendrite. A bipolar theta glass pipette was used for stimulation of the boutons. Changing the stimulation frequency from 0.05 to 1 Hz produced a strong potentiation (>200%) of the GABAergic synaptic responses (black circles). By contrast stimulation of IntBs (open circles) produced frequency-dependent depression. **(B)** The effect of L-AP4 on the pharmacologically isolated GABAergic current evoked by MF bouton stimulation is presynaptic, as evidenced by the paired-pulse protocol. By contrast, GABAergic responses evoked by interneuronal bouton stimulation were unaffected. **(C)** Group synaptic responses to MF bouton (black circles; *n* = 6) and interneuronal bouton stimulation (open circles; *n* = 6). Responses to MF bouton stimulation were partially blocked by iGluR blockers. The remaining responses were strongly and reversibly depressed by the activation of mGluRs with L-AP4, and completely blocked by bicuculline. Responses to interneuronal bouton stimulation were not affected by the perfusion of iGluR blockers, but completely blocked by bicuculline (from Beltrán and Gutiérrez, 2012).

We recently provided what constitutes the most direct evidence in favor of the co-release hypothesis using a preparation that avoids sources of “synaptic contamination.” In dissociated pyramidal cells, with identified boutons of MFs and boutons of interneurons attached to their apical or basal dendrites, we selectively recorded responses to stimulation of each type of bouton (**Figure 3**). Indeed, in young rats stimulation of a number of boutons identified MFs, but not of interneurons, released glutamate, or GABA or co-release glutamate and GABA and these responses had the signature of transmission of MF origin. By contrast, activation of MF boutons of adult rats did not release GABA (**Figure 3**; Beltrán and Gutiérrez, 2012). These data give us a very important message when considering “the existence or not” of co-release: (1) not all boutons release glutamate or GABA or both; altermantively it is not possible to know which cell one records that receives which type of bouton. (2) The number of MF that make contact with postsynaptic cells is very low. Therefore, the possibility of stimulating a fiber (minimal stimulation), finding a responding cell (or doing a paired recording for that matter), which, additionally happens to have co-releasing boutons, points to low odds of everybody doing the experiment in the same way to find the same responses. Therefore, it is the identification of the molecular switch that turns off GABA release during development or turns off (or preventing) GABA release in the adult after seizures that will prove (for all) that GCs are really capable of being GABAergic.

## MOSSY FIBERS EXPRESS GAP JUNCTIONS

**FIGURE 4:**
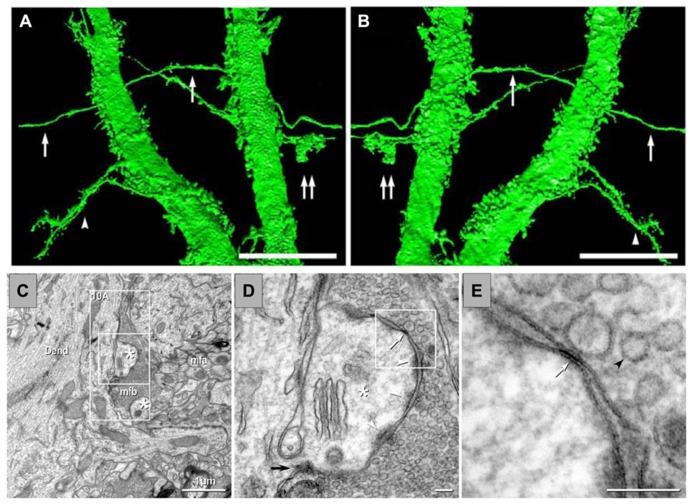
**Anatomical evidence of gap junctions between MFs and pyramidal cell dendrites and thin-section TEM evidence for MF – pyramidal cell mixed synapses. (A,B)** High-resolution 3D reconstructions (in two opposite *z* directions) of two dendrites of a lucifer yellow-injected pyramidal cell, dye-coupled to MFs (arrows). Double arrows mark a MF bouton. The arrowhead in **(C)** marks two dendrites with spines. **(C)** Image showing part of a dendrite (Dend) with dendritic spines (astrisks) and adjacent MFA . **(D)** Higher magnification of boxed area in **(C)**. A typical large-diameter MF bouton containing synaptic vesicles surrounds a dendritic spine at a synaptic contact containing both close membrane appositions characteristic of gap junctions (white arrows) and wider membrane appositions with asymmetric dense cytoplasmic material (arrowheads) characteristic of postsynaptic densities. **(E)** Higher magnification of boxed area in **(D)** showing the gap junction contact (white arrow) between the MF bouton and the dendritic spine. A synaptic vesicle is marked with a black arrowhead (from Hamzei-Sichani et al., 2012).

Hamzei-Sichani et al. (2007) showed the presence of gap junctions on MFs, which suggests that axo-axonal communication between them can underlie very fast network oscillations. Using transmission electron microscopy and freeze-fracture replica immunogold labeling, they were able to determine the presence of Cx36 as the main constituent of these gap junctions. They proposed that only a few gap junctions per axon were present, which contribute to normal axonal electrical activity and that may also be involved in altered electrical activity in epilepsy. Interestingly, not only were the gap junctions found along the MFA themselves but also in the terminals in CA3. Indeed, Hamzei-Sichani et al. (2012) presented ultrastructural, immunocytochemical and dye coupling evidence for mixed electrical-chemical synapses (**Figure 4**). Cx36 was found in MF boutons in contact with dendritic spines of pyramidal cells, among other types of connections. This has also been observed with immunofluorescence and confocal microscopy, whereby Cx36 co-localized with VGLUT1 in the MF boutons (Nagy, 2012).

### EVIDENCE OF ELECTRICAL COMMUNICATION BETWEEN THE MFs

The presence of gap junctions between MFs would enable passage of electrical currents from one axon to the other and, if that is the case, evoking an antidromic action potential in one axon, an electrical spikelet should be seen in another, contiguous cell. Schmitz et al. (2001) provided electrophysiological evidence that coupling potentials were indeed invading a contiguous axon prior to reaching the soma of a nearby cell, and that this effect was blocked by the gap junction blocker carbenoxolone (CBX). This activity provides the basis for an extremely fast communication mechanism and endows the axons with integrative properties, rather than just being a transmission line (Schmitz et al., 2001). Thus, high-frequency network oscillations can have their basis on this type of communication, which allows recruiting principal neurons in a network that generates activity with plastic frequency ranges.

#### Evidence of electrical communication between MFs and pyramidal cells

**FIGURE 5:**
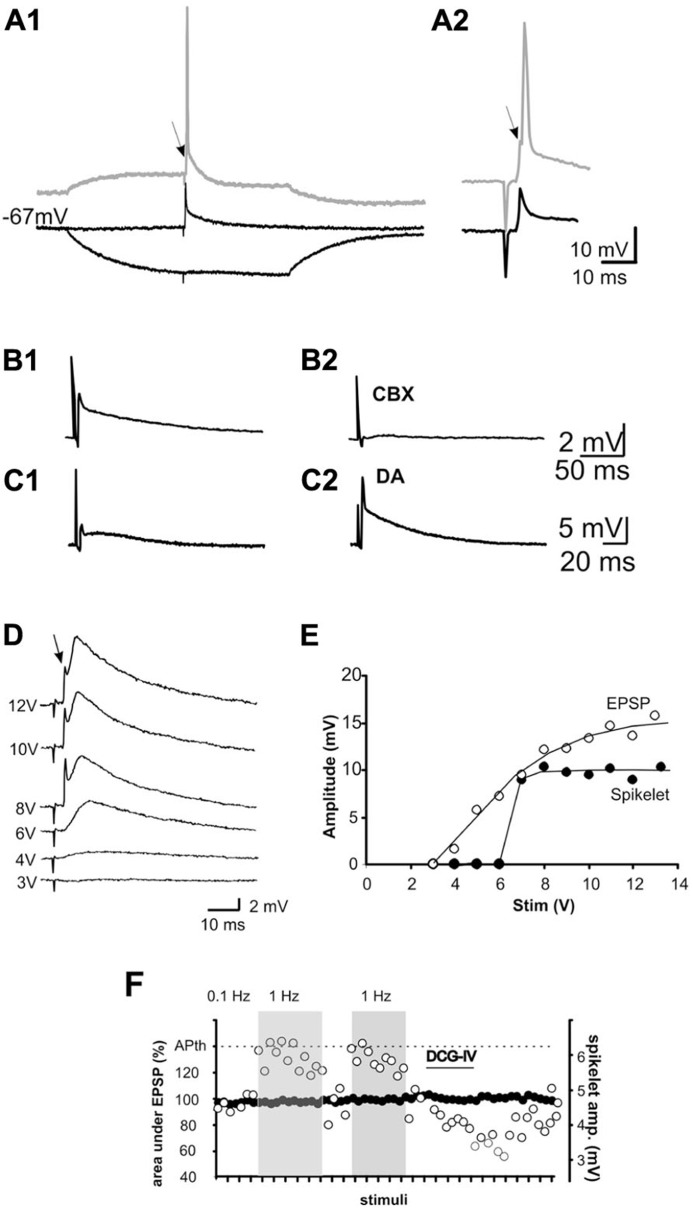
**Mossy fiber activation evokes spikelets in some pyramidal cells (5–7%) of CA3. (A1)** Spikelets are evoked in an all-or-none fashion at resting membrane potential in the presence of glutamate, GABAA and ACh-M1 receptor antagonists. When stimulation is given at a depolarized membrane potential (displaced trace in **A1** and expanded trace in **A2**, the cell fires an action potential, which is preceded by a notch (arrow) corresponding to an underlying spikelet. By contrast, hyperpolarization of the membrane potential by ± 15 mV suppresses the spikelet **B1,2**). The non-selective gap junction blocker, carbenoxolone (CBX) blocked the spikelet, whereas dopamine (DA) potentiated the spikelet **(C1,2)**. If electrical spikelets appear in the MF synapse, a mixed electrical-chemical transmission is expected. This is shown in **(D)**. Increasing stimulation intensities enhanced the chemical component of the synaptic response, whereas the spikelet was evoked upon reaching threshold intensity and not further modified by increasing the stimulus intensity. **(E)** Input–output curve of the chemical (EPSP) and electrical (spikelet) synaptic components. Note that the spikelet is evoked in an all-or-none fashion. **(F)** Plot of the behavior of the EPSP (area under the curve; left *y*-axis) and spikelet (amplitude; right *y*-axis) at 0.01 and 1 Hz and during the perfusion of DCG-IV. APth, action potential threshold.

A hypothesis that can be immediately derived from the observation of Cx36 in MFs is that its expression may not be confined to the axons, but that it can also occur in their terminals in the adult rat. If this were to be the case, MF-to-pyramidal cell electrical communication should be detected. Moreover, the extremely low coupling of MF-to-MF would suggest that also MF-to-pyramidal cell electrical connectivity would be of very low probability, as we also showed to be the case by dye coupling (**Figure 5**; Hamzei-Sichani et al., 2012). Despite the evidence of gap junctions in the principal cells of the DG, axo-dendritic electrical synapses between two different types of principal cells in the hippocampus have not been reported so far. Moreover, no axo-dendritic electrical synapses have been observed between excitatory axons and principal cell dendrites, in the mammalian telencephalon. We provided the first evidence of electrical coupling between MFs and pyramidal cells, which, as expected, were very few (Vivar et al., 2012). Under blockage of ionotropic glutamate and GABA receptors, stimulation of the DG provoked, in about 5% of pyramidal cells recorded (>700), spikelets of fast kinetics, which were blocked by CBX and potentiated by DA. These data are consistent with electrical coupling. Their latency was shorter than of chemical transmission and their MF specificity can be assessed by two criteria. The probability of recruiting interneurons (by electrical synapses), which in turn, make electrical contacts with pyramidal cells is minimal because the delay that we observed is compatible with the conduction velocity of the MFs; by contrast, direct interneuron activation recruiting a pyramidal cell by an electrical contact would produce a response of extremely short latency (<0.4 ms, Mercer et al., 2006); additionally, intensity produced an all or none response, if higher intensities recruit interneurons, latencies would shift strongly (**Figure 5**). Finally, further evidence in favor of this would be given by mixed electrical-chemical transmission.

#### Evidence of mixed electrical-chemical communication between MFs and pyramidal cells. another type of co-transmission of the MFs

As stated above, if electrical communication can be present in a well-known chemical synapse (MF-CA3), then chemical – electrical cotransmission might occur, whereby stimulation of the MFs would evoke an electrical spikelet accompanied by a chemical component. Pharmacological and physiological criteria must then be met to imply that such a response corresponds to transmission of MF origin. We tested this hypothesis and showed that MF activation produced fast spikelets in a number of PCs with an onset latency consistent with MF conduction velocity and were followed by glutamate receptor-mediated excitatory postsynaptic potentials that had the physiological and pharmacological characteristics of MF neurotransmission in the adult rat (Vivar et al., 2012). Moreover, the fast spikelets persisted during blockade of chemical transmission, were potentiated by DA and suppressed by the gap junction blocker CBX. To our knowledge, this constitutes the first evidence of mixed electrical-chemical communication between two principal cells of the mammalian forebrain. We showed that in a number of cells, stimulation of the DG initially produced an EPSP and, when threshold intensity for activating a MF that established an electric synapse was reached, the EPSP was preceded by a fast spikelet. The amplitude of the EPSP augmented as stimulation intensity was increased, whereas the amplitude of the spikelet was not modified. In other cells, spikelets were readily evoked at the minimal intensity needed to evoke any synaptic response. Furthermore, the chemical component of the response was depressed by DCG-IV and was strongly potentiated when raising the stimulation frequency from 0.1 to 1 Hz, while the electrical spikelet remained unaltered (**Figure 5**; Vivar et al., 2012). Mixed electrical and glutamatergic communication between GCs and some pyramidal cells in CA3 may ensure the activation of sets of pyramidal cells, bypassing the strong action of concurrent feed-forward inhibition that GCs activate.

## CONCLUSION

Having reviewed the available data about how the MFs transmit information by different chemicals or by signals of different physical characteristics, one seems to be recollecting the first decades of “modern neuroscience” when “it had to be one or the other.” Or are we still living in those years?

Many features of synaptic communication observed at the MF synapses are not usually observed in most cortical synapses, and thus have drawn the attention of many groups studying different aspects of the transmission of information, of which many authoritative reviews have been published. In recent years, the paradigmatic view that the MFs synapses form merely excitatory, glutamatergic contacts with their post-synaptic target cells has been questioned. In addition, the possibility of them communicating with some of their target cells through gap junctions (Cx36), co-localized with the chemical ones (AMPA-R), compels us to add adjectives to this unique synapse. Short after the first evidence of the co-release of GABA and glycine in the spinal cord (Jonas et al., 1998) and of the possibility that GABA could be co-released with glutamate from the MFs (Gutiérrez and Heinemann, 1997, 2001; Gutiérrez, 2000; Walker et al., 2001) several examples of co-release of classical neurotransmitters in different regions of the CNS appeared (reviewed in Gutiérrez, 2009). Studies on how different transmitters can be released from single synapses ought to be conducted, as some kind of selectivity in the release may be expected, i.e., activity-dependent modifications in content and vesiculation may instruct the synapse to release more of one than the other neurotransmitter. Studies performing quantal release should help to disclose this. Another intriguing possibility is that different presynaptic mechanisms may selectively control the release of either neurotransmitter. As for the post-synaptic site, it is possible that activity-dependent release may evoke receptor incorporation or relocation in the membrane that would tip the balance in favor of a response to one rather than to the other neurotransmitter. These and many questions are still open.

On the other hand, although dendro-dendritic and axo-axonic electrical synapses in the mammalian forebrain are well known, axo-dendritic electrical-chemical synapses were just recently revealed in the MF synapse and there is no doubt that more will certainly appear in different regions of the mammalian CNS. As in the case of the study of the co-release of glutamate and GABA, selective activation of MF boutons should prove true electrical coupling. These experiments, however, are technically difficult because direct stimulation of the bouton can produce an artifact that may obscure the fast electrical component. Despite this, the evidence available is consistent with some MFs establishing electrical coupling with a number of pyramidal cells. This type of contacts in the MF synapse may allow bidirectional, possibly graded communication that can be faster than chemical synapses and which will possibly explain different forms of modulation.

## Conflict of Interest Statement

The authors declare that the research was conducted in the absence of any commercial or financial relationships that could be construed as a potential conflict of interest.
